# Cultural Variations in Evaluation of Creative Work: A Comparison of Russian and Emirati Samples

**DOI:** 10.3389/fpsyg.2021.764213

**Published:** 2021-12-30

**Authors:** Anatoliy V. Kharkhurin, Sergey R. Yagolkovskiy

**Affiliations:** School of Psychology, HSE University, Moscow, Russia

**Keywords:** cultural differences, judgment agreement, structured imagination, implicit theory of creativity, creativity

## Abstract

The study investigates how cultural variations influence evaluation of creative work. Russian and Emirati undergraduate college students were asked to judge alien creature drawings produced by their country mates in previous studies’ structured imagination test. We found cultural differences in creativity judgment. Emirati participants’ judgments were significantly lower than Russian participants’ judgments. We also found that Russians judged their compatriots significantly higher than the Emirati judged their compatriots. Russians also judged foreigners significantly lower than the Emirati judged foreigners. These findings were speculatively placed in the context of the cultural differences in the implicit theory of creativity.

## Introduction

A while ago, the authors participated in the Science Film Festival in Russia where they presented Herman Vaske (2018) documentary “Why Are We Creative? The Centipede’s Dilemma.” In this film, Vaske asks prominent representatives of various creative professions why they are creative. Their answers differed to the extent that virtually no common denominator could have been identified. The film clearly demonstrated that people, even those directly involved in creative professions, seem to be guided by very different notions of creativity.

This phenomenon is addressed in the literature as implicit theory of creativity, which is traditionally contrasted with explicit theory of creativity. The latter usually reflects the scientific study of a concept. Sternberg (1985, p. 607) defined explicit theories as “constructions of psychologists or other scientists that are based on or at least tested on data collected from people performing tasks presumed to measure psychological functioning.” These theories are developed by professional scholars and shared through academic and semi-academic venues, such as journals, conferences, and talk shows (Runco et al., 1998).

In contrast, implicit theories or folk conceptions refer to constructs tacitly presented in people’s minds regardless of their expertise. These are sets of beliefs shared by a sociocultural group about the world, such as folk concepts of intentionality (Malle and Knobe, 1997), lay epistemics (Kruglanski, 1989), or implicit personality theories (Schneider, 1973). These are “the ideas held by laypeople that are usually not discussed, questioned, or consciously considered” (Paletz and Peng, 2008, p. 288). As became evident from Vaske’s documentary, people hold very different opinions about the construct of creativity and consequently experience difficulties formalizing it. Therefore, an in-depth examination of implicit theories of creativity can help scholars obtain a more realistic and practical opinion about the construct (Runco and Bahleda, 1986).

The question is how the implicit theory of creativity can be studied empirically. To answer this question, we need to understand how it is manifested in different facets of people’s activity. Paletz et al. (2011) specified two ways in which people can express their implicit theory of creativity: externally (through interaction with others and generation of various products) and internally (by means of personal and inner processes). The external manner of expressing implicit theory of creativity could be addressed by looking at how creative production and performance is influenced by people’s tacit conception of creativity. The internal approach develops in two directions. Some studies focused on how the implicit theory of creativity is realized in people’s evaluation and assessment of their own and other people’s creative abilities and personality traits (e.g., Lim and Plucker, 2001; Pizzingrilli and Antonietti, 2010; Hass, 2014; Stone and Hess, 2020). Others looked at how the implicit theory of creativity influenced people’s judgment of creative work produced by others (e.g., Niu and Sternberg, 2001; Storme and Lubart, 2012; Long, 2014; Benedek et al., 2016; Loewenstein and Mueller, 2016; Stemler and Kaufman, 2020). The latter appears to be an important topic for investigation considering that people’s subjective judgment of creative work could play a fatal role in one’s creative aspirations and career. A number of studies demonstrated that one’s career and creative achievements are predicted by self-efficacy (e.g., Tierney and Farmer, 2002; Baer et al., 2008), which in turn, is related to the judgment of one’s creative works and creative capacity (e.g., Bandura, 1997; Jaussi et al., 2007).

All these studies address the concept of implicit theory of creativity indirectly. That is, they assume the manifestation of the implicit theory in creative production and performance as well as evaluation of creative work produced by oneself and the others. The present study is grounded in the assumption that the evaluation of creative work of others is influenced by the implicit theory of creativity. Note that we do not intend to test this assumption empirically. Rather, we look at its derivation, namely, evaluation of creative work produced by the others, and cultural variations thereof. In doing so, we employ a judgment paradigm.

The judgment paradigm is widely used in both creative enterprises and creativity research. Expert judges form juries at film festivals, art fairs, and musical contests. Guided by their expertise, they provide a consensual opinion about the creative value of a product and/or performance. In empirical research, Amabile (1982) developed Creativity Assessment Technique, which relies on the consensual assessment of creative production by several judges. This technique is widely used in creativity research (e.g., Kaufman et al., 2008; Baer and McKool, 2009; Freeman et al., 2015; Stefanic and Randles, 2015; see overview in Barth and Stadtmann, 2020). At the same time, Amabile pointed out that “These studies, too, often suffer from a definitional void by failing to explicitly articulate the definition of creativity…” (Amabile, 1982, p. 1000). Research shows that individuals’ judgment of creative work varies due to their own level of creativity (e.g., Caroff and Besançon, 2008; Guo et al., 2019) and their expertise (e.g., Kaufman et al., 2009, 2013; Plucker et al., 2009). Hence, we should expect a variation in creativity judgment between experts and laypeople. The former group was well studied, and Creativity Assessment Technique was widely used as an assessment of creativity in different domains (e.g., Yuan and Lee, 2014; Daly et al., 2016; see overview in Cseh and Jeffries, 2019). The latter, however, did not receive sufficient attention. Our study focuses on laypeople who have neither expertise nor experience in creative enterprise. Therefore, their judgment can be assumed to be influenced by their implicit theory of creativity.

Contemporary creativity literature suggests that cultural aspects of the environment have considerable influence both on levels of creative potential and on how creativity is evaluated (e.g., Simonton, 1994; Lubart and Sternberg, 1998; Niu and Sternberg, 2001; Glăveanu, 2010; Glăveanu et al., 2014). Cultural psychologists often describe culture as a set of beliefs, moral norms, customs, practices, and social behaviors of a particular nation or a group of people whose shared beliefs and practices identify the particular place, class, or time to which they belong (e.g., Rohner, 1984; Peng et al., 2001; Paletz and Peng, 2008). A set of common mental models, cultural scripts, and “interpretive frames” (Pavlenko, 2000) characterizes these people and suggests strategies in solving problems and dealing with a variety of situations in a culture-specific way. Cultural values and norms are assumed to determine and shape the concept of creativity, which in turn may influence the manner in which creative potential is apprehended and incarnated (e.g., Rudowicz, 2003; Westwood and Low, 2003; Lubart, 2010). There is even a radical opinion that “no account of creativity can be satisfactory unless it is culture-inclusive” (Glăveanu, 2010, p. 151).

Hence, we should expect cultural variations in people’s perception of the concept of creativity. That is, people in the same cultural group tend to share some defining aspects of creativity, whereas people from different cultural groups tend to differ in some defining aspects of creativity. This can be illustrated with a distinction in the view of creativity in the West and in the East (e.g., Niu, 2019). Literature distinguishes between the West and the East with respect to individualism and collectivism (Triandis, 1975, 1977) or with respect to an independent and interdependent perspective (Markus and Kitayama, 1991). The distinction between these two social systems is grounded upon the degree of subordination of an individual’s personal goals to the goals of some collective (Triandis et al., 1985). The individualist society values the person’s unique qualities, initiative, and achievement, whereas the collectivist one places more emphasis on consensus with the community, on being in line with the others. This distinction manifests itself in different perceptions of creativity.

Within the tradition of Western psychology, creativity is understood as a factor, which determines the generation of a novel and appropriate ideas or solutions to a problem. It is closely related to originality (novelty) and usefulness (appropriateness; see Sternberg, 1999, for an overview). By analyzing the anthropological and philosophical literature on creativity in Indian, East Asian, and African societies, Lubart (1990, 1999) revealed distinct Eastern and Western conceptions of creativity: Western concept of creativity is understood as having a finite beginning and end; in contrast, Eastern understanding of creativity supposes development. So, Western understanding of creativity emphasizes innovation, whereas Eastern concept is more dynamic assuming creative people’s ability to reuse and reinterpret existing traditions (Lubart, 1999; Raina, 1999; see overview in Paletz and Peng, 2008). For example, contemporary Western art appreciates novelty and radical changes in existing paradigms or even rejection of them. In contrast, Confucian esthetics is related to the re-consideration of existing ideas, which reflects own values and beliefs (Tu, 1985). That is the case of traditional Arabic calligraphy or Chinese brush painting: the old ideas could be modified to reflect an artist’s authentic perception. The latter could be regarded as a creative tool that is capable of catching the essence of the object. Instead of trying to establish a unique phenomenon by breaking up with old traditions, a person cultivates one’s authentic approach, which can be applied to both old and new (Averill et al., 2001).

If representatives of different cultures may differ in their perception of the creativity construct, these differences may influence the evaluation of creative work produced by other people. A few cross-cultural studies investigated the agreement on creativity ratings of the work produced by the others. Participants were asked to judge creative work produced by other people. Chen et al. (2002) had American and Chinese college students evaluate drawings produced by their respective peers based on geometric shapes (circle, rectangle, and triangle). The agreement between the US and Chinese judges was nearly perfect (overall correlation was 0.97). Niu and Sternberg (2001) asked US and Chinese graduate students in psychology to make collages and to draw an alien creature (cf. Ward, 1994). Then, they asked the US and Chinese judges to evaluate these works. Americans were found to produce more creative artworks than did their Chinese peers, and this performance difference was recognized by both American and Chinese judges. Moreover, the difference between the use of criteria by American and Chinese judges was small. Very similar findings were reported in a study comparing German and Chinese participants’ performance on occupational creative problem-solving task (Tang et al., 2015). The task performance was evaluated using the Consensual Assessment Technique mentioned above with judges from respective countries. That study revealed that both German and Chinese judges rated the German respondents’ outcomes higher on most creativity dimensions. In a similar vein, Yi et al. (2013) reported that both German and Chinese judges found that German participants produced more creative and esthetically pleasing artwork than did their Chinese counterparts.

### The Present Study

The present study takes a similar approach and looks at how cultural variations may influence the evaluation of creative work produced by other people.

We employed samples from Russia and the United Arab Emirates (UAE). Traditionally, the Western conception of creativity is ascribed to people from North America and Western Europe (e.g., Niu and Sternberg, 2001; Yi et al., 2013; Tang et al., 2015; Loewenstein and Mueller, 2016). A few studies demonstrated that people from Eastern and Central Europe also tend to reveal Western perspective on the creativity construct (e.g., Glăveanu and Karwowski, 2013; Hojbotă, 2013; Pavlović et al., 2013; Szen-Ziemiańska, 2013). At the same time, scientific literature lacks research on perception of creativity among Russians, although they appear to share a mindset with those residing in Central and Eastern Europe and there is an argument that Russia is inclined toward a more Western way of thinking (see discussion below). In a similar vein, the Eastern perspective on creativity is largely represented by Asian countries (specifically, China, see discussion above) and underrepresented by Middle Eastern countries. Hence, it appears to be plausible to test samples from the countries that are underrepresented in the literature.

Thus, we selected Russia as a representative of a European country (more West oriented) and the UAE as a representative of the Middle Eastern country (more East oriented). This selection is supported by the literature demonstrating Russia’s rapid transition toward a less collectivist and more democratic society (e.g., Stetsenko et al., 1995; Ryan et al., 1999; Naumov and Puffer, 2000). The UAE, on the other hand, is considered a traditional collectivist Middle Eastern society (e.g., Hameed et al., 2016; Rao et al., 2021).

Kharkhurin and Yagolkovskiy (2019) and Kharkhurin and Charkhabi (2021) used structured imagination test (Ward, 1994) to collect drawings of an alien creature from Russian and Emirati participants, respectively. The test evaluated participants’ ability to surpass their “structured imagination” (cf. Ward, 1994), which presumably limits individuals’ thinking outside the box. That is, people have difficulties violating the conceptual boundaries of a standard category when creating a new exemplar of that category. The drawings of the alien creature obtained from Russian and Emirati participants in those studies were judged by a different group of Russian and Emirati participants in the current study.

Considering reviewed evidence, we advanced a hypothesis that taps into cultural variations in creativity judgment. We expected to find that representatives of different cultural groups judge creative work produced by the others differently.

## Materials and Methods

### Procedure

The study consisted of two phases. Phase I involved selection of drawings of an alien creature produced in the test of structured imagination (see description below). These drawings were randomly selected from the ones produced in Russia and reported by Kharkhurin and Yagolkovskiy (2019) and in the UAE and reported by Kharkhurin and Charkhabi (2021), respectively. To save space in the present article, we refer the reader to those studies for a detailed description of the methods and present here only the information relevant to the purpose of the present study, namely, the description of the randomly selected samples and the test of structured imagination.

Thus, in Phase I, we formed a pool of 100 drawings of alien creatures: 50 drawings were randomly selected from the Russian sample and another 50 were randomly selected from the UAE sample. To eliminate any language related bias, the drawings were cleaned from any text using Adobe Photoshop CS5.1.

A total of 100 drawings of alien creatures were used in Phase II’s creativity judgment procedure (see description below). They were presented using the open-source survey platform LimeSurvey 2.06. The order of presentation was random.

In Phase II, after signing the consent form, participants received the creativity judgment procedure.

### Participants

Participants from Russia and the UAE were recruited in both phases of the study. To reduce potential sampling biases, we recruited undergraduate students from the highly reputable Universities in the respective regions. HSE University was ranked ninth in Russia and the American University of Sharjah was ranked seventh in the Middle East according to QS World University Rankings.^1^

In Phase II (current study), the Russian sample consisted of 53 (13 male and 40 female) HSE University (Russia) undergraduate students aged between 17 and 20 (*M* = 18.94, *SD* = 1.05). The UAE sample consisted of 53 (15 male and 38 female) American University of Sharjah (UAE) undergraduate students aged between 17 and 26 (*M* = 20.04, *SD* = 1.78). Participants were invited to participate in the study through the Introduction to Psychology subject pool powered by the SONA systems.^2^ They received a course credit for participation in the study.

The drawings selected in Phase I (previous studies) were produced by 50 (20 male and 30 female) HSE University (Russia) undergraduate students aged between 17 and 21 (*M* = 18.18, *SD* = 0.75), who were randomly selected from a sample used by Kharkhurin and Yagolkovskiy (2019) and by 50 (20 male and 30 female) American University of Sharjah (UAE) undergraduate students aged between 17 and 23 (*M* = 20.10, *SD* = 1.42), who were randomly selected from a sample used by Kharkhurin and Charkhabi (2021).

### Instruments

Emirati participants received all tests in English and Russian participants received all tests in Russian. The Russian versions of the tests were produced from the original English versions using back-translation (Brislin, 1970).

#### The Test of Structured Imagination

Structured imagination was assessed using modified version of the Invented Alien Creatures task (cf. Ward, 1994; Kozbelt and Durmysheva, 2007). The task was reduced from the original version to suit the purpose of the present study. The participants were asked to imagine, draw, and describe a creature living on a planet very different from Earth. They were encouraged to be as imaginative and creative as possible and not to worry about how well or poorly they draw. They had 12 minutes to complete the task.

An invariant coding system (Kozbelt and Durmysheva, 2007) was used to categorize each drawing on three *invariants*, the features that commonly appear in most participants’ responses: bilateral symmetry, two eyes, and four limbs. The chosen invariants were similar to the ones extracted in Ward’s (1994, p. 1) original study, in which he found that “the majority of imagined creatures were structured by properties that are typical of animals on earth: bilateral symmetry, sensory receptors, and appendages.”

**Figure 1:**
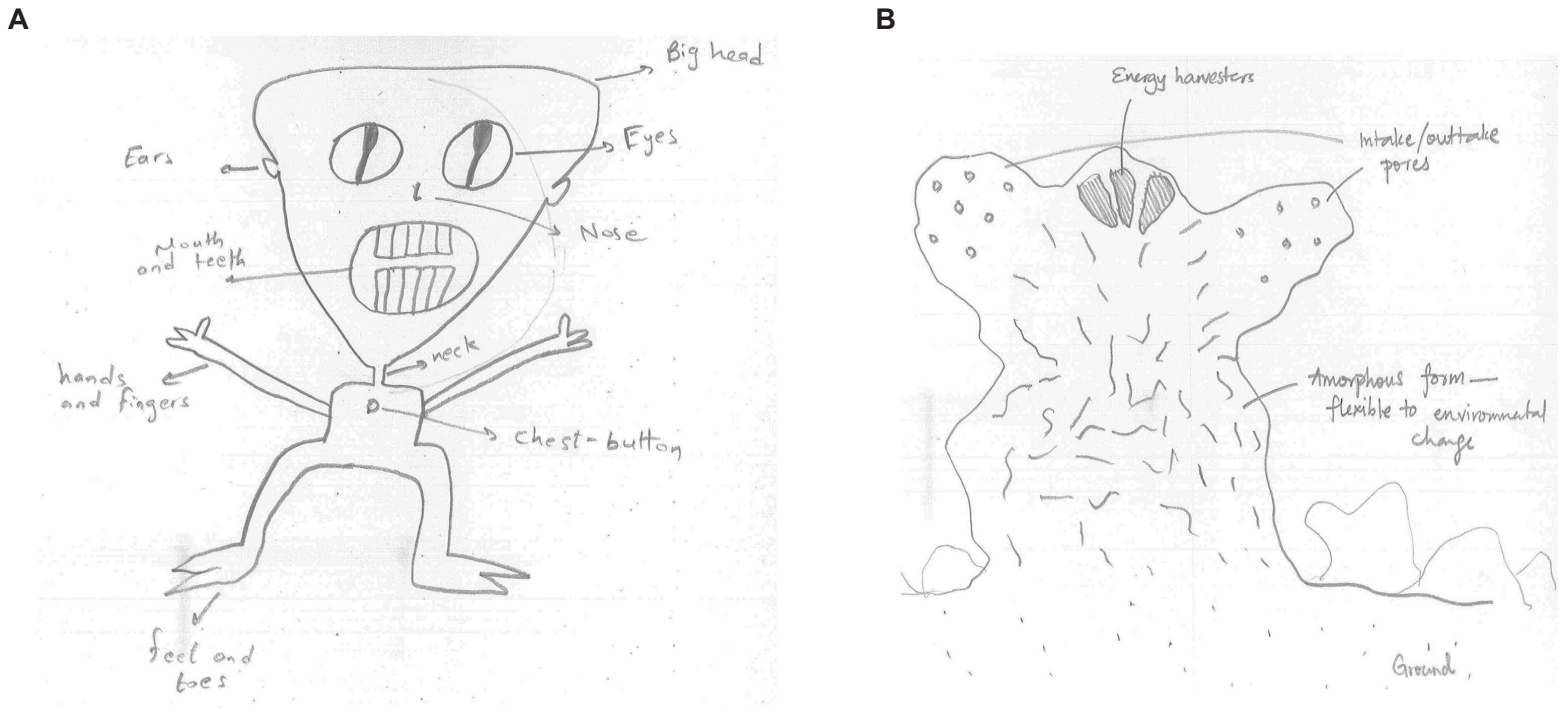
Examples of the alien creatures produced in the Invented Alien Creatures Task, which received **(A)** low (0) and **(B)** high (4) invariant violation scores, respectively.

Each invariant had five categories each of which was assigned a value indicated in parentheses. For bilateral symmetry, the categories were: clearly bilaterally symmetric (0), bilaterally symmetric if the creature was rotated (0), superficially violating bilateral symmetry (e.g., an extra limb on one side; 1), clearly not bilaterally symmetric (2), and unclear (0). For eyes and limbs, the categories were as: clearly following the invariant (two eyes or four limbs; 0), drawing more features than the invariant (more than two eyes or four limbs; 2), drawing fewer features (one eye or one to three limbs; 2), drawing no relevant features (1), and unclear (0). So, each drawing received an invariant value ranging from 0 (not violated) to 2 (clearly violated). Subsequently, the total *invariants violation* score was calculated as a sum of three invariants scores. The invariants violation score ranged from 0 to 6; a higher score suggested a greater tendency to violate the standard invariants in the drawing. Figure 1 presents two cases (before language related material was removed) illustrating (a) no violation of invariants and (b) some violation of invariants. The creature in Figure 1A is bilaterally symmetric (score 0), has two eyes (score 0) and four limbs (score 0); its invariants violation score is 0. The creature in Figure 1B is bilaterally symmetric (score 0), has more than two eyes (score 2) and no limbs (score 2); its invariants violation score is 4. The invariants violation score was used in the present study as an indicator of *creative production*.

#### Creativity Judgment Procedure

Participants received 100 drawings of alien creatures (see Procedure above). They were asked to judge the level of creativity of each drawing and indicate it on a five-point Likert-type scale. A higher *creativity judgment* score indicated greater perceived creativity of the drawing.

## Results

**Figure 2:**
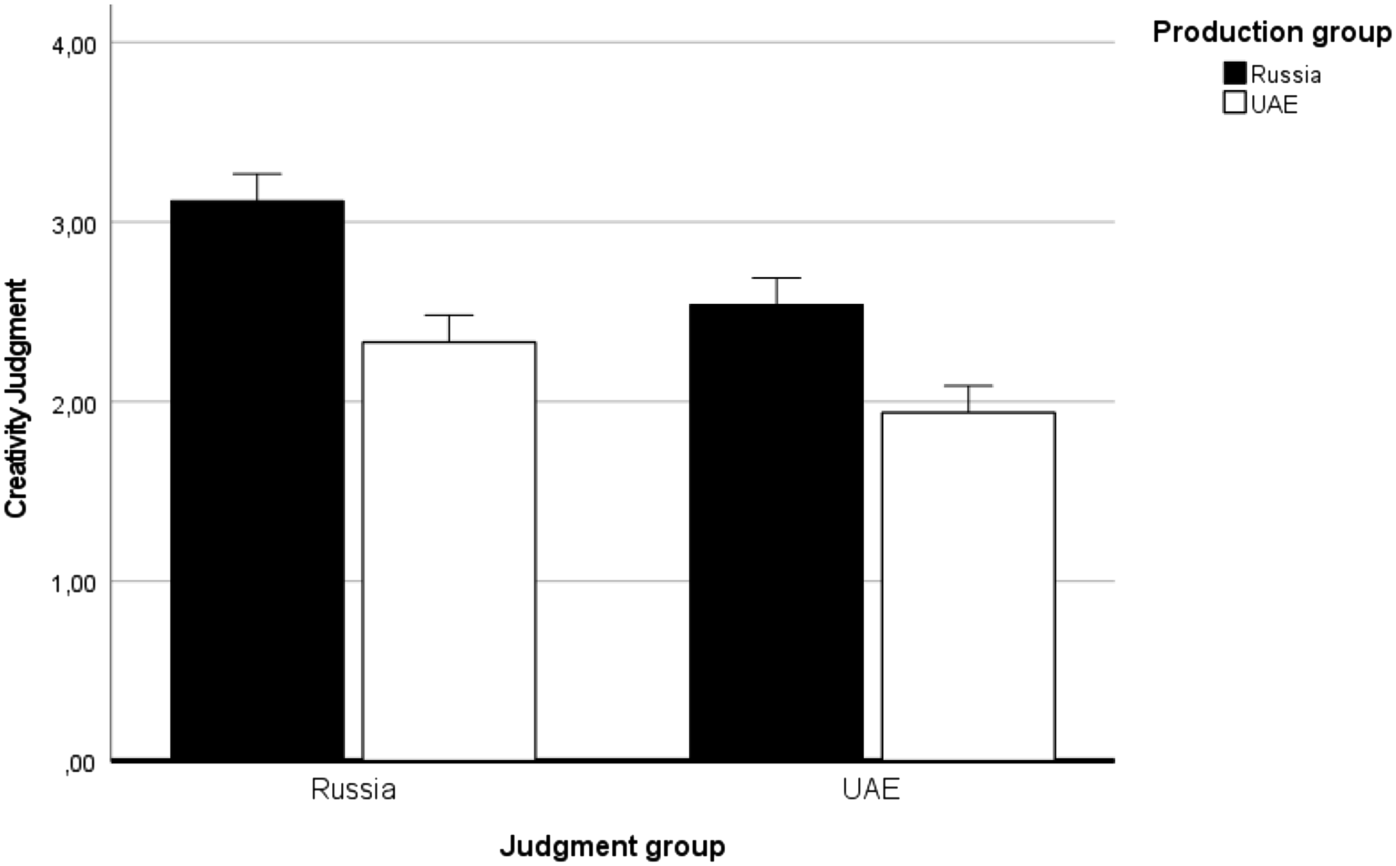
The Russian and Emirati participants’ judgments (Phase II) of the Invented Alien Creature drawings produced by Russian and Emirati participants (Phase I).

For each alien creature drawing produced in Phase I, we separately calculated a mean judgment score produced by Russian and Emirati participants in Phase II. A rate of agreement between Russian and Emirati judgment scores was high (*r* = 0.89, *p* < 0.001). Table 1 and Figure 2 present mean judgment scores obtained from Russian and Emirati participants (Phase II, judgment) of the drawings produced by Russian and Emirati participants (Phase I, production). From now on, we call cultural groups in Phase I drawing *production* groups and those in Phase II – drawing *judgment* groups.

**Table 1:** Mean judgment scores by each judgment group (Phase II) for the alien creature drawings produced by each production group (Phase I).

Drawing judged by	Russia	UAE
**Drawing produced by**		
Russia	3.12 (0.52)	2.54 (0.58)
UAE	2.33 (0.51)	1.94 (0.47)

As Figure 2 demonstrates, the Russian judgment group gave higher ratings to both production groups in comparison with their Emirati peers. Also, both judgment groups gave higher ratings to the Russian production group than to the Emirati production group. These observations were tested in the following analysis.

Note that we found an effect of cultural groups on drawing production. That is, in the Phase I, Russian production group obtained significantly greater invariants violation scores than the Emirati production group [*ΔM* = 0.62, *SE* = 0.27, *t*(98) = 2.53, *p* < 0.05]. Since the production groups differed in their invariants violation scores, its potential confounding effect should be taken into account. In other words, to ensure that the differences in cultural groups’ judgments were not stipulated by the differences in the quality of the drawings produced by respective cultural groups, we controlled the potential effect of the latter.

Thus, we performed a repeated-measure ANCOVA with the drawing production group (Russia, UAE) as between-subject factor, the drawing judgment group (Russia, UAE) as within-subject factor, and the invariants violation score as a covariate. The multivariate analysis with Wilks’ Lambda revealed a main effect of the drawing judgment group [*Λ* = 0.54, *F*(1, 97) = 82.31, *p* < 0.001, *η*^2^ = 0.46]. The Russians’ judgments were significantly higher than the Emirati’s judgments [*ΔM* = 0.48, *SE* = 0.03, paired *t*(99) = 4.52, *p* < 0.001]. The test of between-subjects effects also revealed a significant effect of the drawing production group [*F*(1, 97) = 41.41, *p* < 0.001, *η*^2^ = 0.30]. Overall, the judgment scores for the Russian production group were significantly higher than the judgment scores for the Emirati production group [*ΔM* = 0.67, *SE* = 0.05, paired *t*(105) = 15.30, *p* < 0.05 when adjusted by Bonferroni correction]. Finally, in the multivariate analysis with Wilks’ Lambda, we found an interaction effect between the production and judgment groups on judgment [*Λ* = 0.92, *F*(1, 97) = 8.04, *p* < 0.01, *η*^2^ = 0.08]. Judgment of compatriots by the Russian group was significantly higher than by the Emirati group [*ΔM* = 1.18, *SE* = 0.10, paired *t*(49) = 12.30, *p* < 0.001], whereas judgment of foreigners by the Russian group was significantly lower than by the Emirati group [*ΔM* = −0.21, *SE* = 0.09, paired *t*(49) = −2.25, *p* < 0.05]. However, as Figure 2 clearly shows, the former difference was much greater than the latter.

## Discussion

We explored whether cultural variations influence perception of creative merit in the work produced by the others. Our hypothesis that the representatives of different cultural groups judge creative work differently was confirmed. The main finding demonstrated variations in the cultural groups’ judgment of the work produced by the others. Emirati participants’ judgments were significantly lower than Russian participants’ judgments.

Why did the Emirati tend to rate the alien creature drawings lower than the Russians? A possible answer to this question taps into perception of the creative value of the violation of standard category boundaries as assessed by the structured imagination test. Recall our finding that in the structured imagination test presented in Phase I of the study, Emirati participants tended to violate invariants less likely than their Russian counterparts. The reluctance to violate invariants in creative production can be related to disfavoring the creative work violating those invariants. That is, Emirati participants may find the drawings with more invariant violation less attractive and judge them as less creative. This idea is supported by an additional analysis demonstrating that invariant violation scores obtained in Phase I correlated significantly with Russian judgment scores (*df* = 48, *r* = 0.26, *p* < 0.05) and insignificantly with Emirati judgment scores (*df* = 48, *r* = 0.18, *p* = 0.07). Thus, Emirati participants may perceive the drawings that violate invariants as less creative, whereas Russian participants may perceive those drawings as more creative.

The violation of invariants in structured imagination test (Ward, 1994) presumes violating the conceptual boundaries of a standard category when creating a new exemplar of that category. In general, unstructured imagination, thinking outside the box is considered to be an important criterion of creative thinking. People rate the drawings of the alien creatures that revealed more violations of a standard set of properties characterizing a category as more creative (e.g., Marsh et al., 1996; Kozbelt and Durmysheva, 2007; Kharkhurin, 2009). However, it is entirely possible that unstructured imagination appears to be a defining property of creativity in the Western, but not in the Eastern tradition.

Kharkhurin (2014) claimed that the Western creative tradition places more emphasis on novelty and originality in thinking, whereas the Eastern tradition values esthetics, goodness, and authenticity. Li (1997) distinguished between horizontal and vertical traditions in the production of art. According to the former (typical for Western cultures), the symbols, methods, and aims of art are subject to modification and even radical change. In contrast, the latter tradition (more characteristic of Eastern cultures) constrains both the content and the techniques of the artistic work and places more emphasis on the esthetic values of the product. Hence, the Western perspective on creativity encourages violation of standards, whereas the Eastern perspective assumes the conformity to the standard. In the present study, Russians guided by the Western creative tradition may value unstructured imagination more than Emirati. As a result, the Russian participants produced higher judgment scores than their Emirati counterparts.

Further, similar to the above mentioned cross-cultural studies of the judgment agreement (Niu and Sternberg, 2001; Chen et al., 2002; Yi et al., 2013; Tang et al., 2015), we found a high rate of agreement between the Russian and Emirati participants evaluating the alien creature drawings. Both cultural groups gave higher rating scores to the drawings produced by the Russians. A possible explanation of this agreement is rooted in compatibility of evaluation criteria used by both cultural groups. This idea was expressed by Haritos-Fatouros and Child (1977) who found a consistency in evaluation of esthetic qualities of artwork by American and Greek judges. Interpreting their findings, they proposed that judges from different countries use similar criteria in their assessment of artworks, and esthetic components of these judgments have transcultural stability. Note that we cannot make a parallel between that study and ours, because Greek and American cultural settings appear to be closer to each other than the Russian and the Emirati ones. However, we appreciate Haritos-Fatouros and Child’s conclusion about the transcultural stability of esthetic components of creative work.

However, some scholars provide a counter argument claiming that people tend to reproduce their own cultural systems in their artistic expression and evaluation of artwork (e.g., Shweder, 1991; Morling and Lamoreaux, 2008). This argument was supported by a number of studies demonstrating that people prefer to judge creative work produced by their country mates higher than the one produced by the foreigners (e.g., Wang et al., 2012; Ishii et al., 2014; Bao et al., 2016). For example, Bao et al. (2016) presented Chinese and international students from Western countries with traditional Chinese paintings and Western classicist paintings. They found a significant interaction between the cultural origin of the painting and the cultural background of the judges. Western participants rated Western paintings higher than Chinese paintings, whereas Chinese participants evaluated traditional Chinese paintings higher compared to Western paintings. Similarly, Ishii et al. (2014) revealed differences between European Americans and Japanese participants in the preference for unique and harmonious colorings. Wang et al. (2012) found differences between East Asians and European Canadians in their preferences for web page complexity.

An additional finding of the present study revealed that the Russian participants judged the drawings produced by their country mates as more creative than those produced by the foreigners, whereas the Emirati judged the drawings of their country mates as less creative than those produced by the foreigners. An obvious explanation of this finding can be inferred from the cultural differences in performances on structured imagination test and judgment procedure. The Russian participants in Phase I obtained higher invariant violation scores than their Emirati peers. At the same time, the Emirati participants in Phase II tended to provide lower creativity judgment scores than the Russian peers. Hence, lower ratings by the Emirati judgment group of the lower performance of the Emirati production group resulted in the lowest judgment scores by the Emirati judgment group of the Emirati production group.

## Conclusion

In conclusion, we would like to place our findings in a broader context pertinent to contemporary research in creativity as well as to potential developments of the project.

First, we consider the methodological implications of our findings. The present study demonstrated that culture-specific variations may have an impact on the evaluation of creative work. As was stated in the introduction, culture-specific mental models and interpretive frames suggest common strategies in solving creative problems and thereby influence creative behavior. Our study demonstrated that these cultural aspects may also create a bias in the evaluation of creative performance of the representatives of alien cultural groups. Although this issue has not received sufficient coverage in empirical research, it appears to be quite an urgent matter. For example, Kharkhurin (2014) argued that the Western creative tradition places more emphasis on novelty and originality in thinking. In contrast, in the manifestation of creative abilities in the Eastern tradition, esthetics (e.g., Kay, 1996; Zuo, 1998), goodness (e.g., Chan, 1967; Lao, 1983; Lao-Tzu, 1992), and authenticity (e.g., Tu, 1985; Averill et al., 2001) rather than originality play a pervasive role. Most of the existing assessment techniques adopted a Western construct of creativity, which emphasizes originality in thinking. Therefore, they could be biased toward typical Western creative behavior and disregard creative principles inherent to non-Western cultural groups. This bias in creativity assessment could explain the empirical findings of the Western dominance pervasive in creativity research.

Second, we would like to go back to our initial idea and place our findings of cultural differences in judgment in the context of the implicit theory of creativity. Professionals in creative domains base their evaluation of a creative product on specific knowledge and expertise, i.e., on explicit theory of creativity. In contrast, lay people tend to use popular knowledge about creativity without necessarily precise specifications, i.e., implicit theory of creativity. These people’s judgments are instructed by their own (often naïve) understanding of the concept of creativity. This is the case of our study’s participants. Therefore, we could suppose that the judgment of the alien creature drawings in the present study was guided by participants’ implicit theories of creativity. In other words, there is a link between implicit theory of creativity and judgment of creative work. Our study revealed that the specifics of the sociocultural environment could have an impact on the judgment of creative products. Hence, we could make an inference that sociocultural context may influence implicit theory of creativity. This assumption finds support in the literature demonstrating cultural effects on the implicit theory of creativity (e.g., Lim and Plucker, 2001; Paletz and Peng, 2008; Lan and Kaufman, 2012).

Third, we extend our considerations about the implicit theory of creativity to a newly developed conception of creative perception. Let us imagine ourselves mingling with a museum crowd. It is implicit rather than explicit theory of creativity that instructs our opinion about the merit of exhibited artworks. Variations in implicit theory of creativity influence not only how creativity is incarnated, but also how it is appreciated. Our evaluation of a creative product is instructed by our perception of the construct of creativity. This idea brings us to an arising creative perception paradigm. In a first sketch of this paradigm, Kharkhurin and Charkhabi (2021, p. 10) proposed that “creative perception can be defined as an individual’s ability to identify creative elements in oneself, others, and the environment.” These creative elements appear to be essential constituents of phenomenal reality that reflect the fundamental truth of nature (Kharkhurin, 2014). The ability to identify these elements encourages an individual to engage in the process of expressing them in one’s creative act. In fact, perception of creativity construct refers to implicit theory of creativity. Creative perception may not only facilitate expression of phenomenal reality in a creative work. It can also instruct the beholder’s perception of the creative work produced by the others. This is a well-known phenomenon in contemporary art. One needs to be prepared (and even educated) to appreciate an artwork.

Finally, we would like to propose several directions for future research. Cultural variations in implicit theory of creativity imply that people with different cultural backgrounds may vary in their explicit definitions of creativity. It would be interesting to see how those distinctions reflect the differences in creative perception, production, or judgment? A plausible methodology would analyze the definitions of creativity provided by the representatives of different cultural groups and relate them to their performance on various assessments of creativity. This could be a theme for the next study. Another study could explore the specific criteria used by people from different cultural groups to evaluate creative work produced by the others.

## Data Availability Statement

The raw data supporting the conclusions of this article will be made available by the authors, without undue reservation.

## Ethics Statement

The studies involving human participants were reviewed and approved by the Institutional Review Board of American University of Sharjah. The participants provided their written informed consent to participate in this study.

## Author Contributions

AK designed the study, collected data in UAE, and drafted the manuscript. SY collected data in Russia and edited the manuscript. All authors contributed to the article and approved the submitted version.

## Funding

The article was prepared in the framework of a research grant funded by the Ministry of Science and Higher Education of the Russian Federation (grant ID: 075-15-2020-928).

## Conflict of Interest

The authors declare that the research was conducted in the absence of any commercial or financial relationships that could be construed as a potential conflict of interest.

## Publisher’s Note

All claims expressed in this article are solely those of the authors and do not necessarily represent those of their affiliated organizations, or those of the publisher, the editors and the reviewers. Any product that may be evaluated in this article, or claim that may be made by its manufacturer, is not guaranteed or endorsed by the publisher.

## Footnotes

^1^ http://www.topuniversities.com/

^2^ https://www.sona-systems.com/
